# Radiation-Induced Urologic Complications: A Case Report on Managing Hydronephrosis and Recurrent Urosepsis

**DOI:** 10.51894/001c.144616

**Published:** 2025-09-30

**Authors:** Olorunferanmi Oni

**Affiliations:** 1 Department of Internal Medicine McLaren Greater Lansing

## 107

### INTRODUCTION

Pelvic radiation therapy is essential for cancer treatment, but it can lead to long-term complications. Although radiation-induced ureteral fibrosis and hydronephrosis are known complications, persistent, recurrent urosepsis over a decade is uncommon. This case compellingly demonstrates an uncommon case progression of post-radiation complications that challenges conventional management. It highlights the difficulty of treating chronic UTIs and multidrug-resistant infections in a patient requiring long-term ureteral stenting, emphasizing the need for further clinical insight, innovative management strategies, and a multidisciplinary approach in complex post-radiation cases.

### CASE DESCRIPTION

A 60-year-old female with stage III endometrial cancer underwent a total abdominal hysterectomy with bilateral salpingo-oophorectomy in 2007, followed by EBRT and chemotherapy. By 2009, she developed radiation-induced right-sided hydronephrosis, requiring ureteral stent placement with exchanges every three months. From 2021, she experienced recurrent septic episodes and acute kidney injury due to Proteus species UTIs. A CT scan revealed bilateral hydronephrosis and a new left ureteral stone, prompting emergent cystoscopy and bilateral stenting.

Between 2022 and 2024, she required multiple hospitalizations for MDRO-related bacteremia, including Klebsiella pneumoniae, E. coli, vancomycin-resistant Enterococcus (VRE), MRSE, Pseudomonas putida, and Candida glabrata. Despite prophylactic antibiotics and regular stent exchanges, she continues to experience recurrent infections.

### DISCUSSION

Despite regular stent exchanges and prophylactic antibiotic use, the patient continues to experience recurrent septic episodes, highlighting the persistent risk of bacterial colonization and biofilm formation on indwelling urinary devices. The emergence of highly resistant pathogens further complicated management and limited antibiotic treatment options.

Definitive surgical interventions, such as ureteral reconstruction or urinary diversion, were considered but deemed unsuitable due to the patient’s significant comorbidities, including metabolic syndrome, peripheral vascular disease, and a history of DVT/PE. Nephrostomy tube placement, a potential alternative for urinary drainage, was also deferred due to personal considerations, illustrating the importance of shared decision-making in complex cases.

This case reinforces the need for a multidisciplinary approach, integrating urology, infectious disease, nephrology, and palliative care to balance urinary drainage, infection control, and quality of life. Future research should focus on optimizing urinary drainage and developing novel antimicrobial strategies to improve long-term outcomes for patients with radiation-related urologic complications.

